# Kinetics of Azanone (HNO) Reactions with Thiols: Effect of pH

**DOI:** 10.1007/s12013-021-00986-x

**Published:** 2021-05-05

**Authors:** Renata Smulik-Izydorczyk, Karolina Dębowska, Michał Rostkowski, Jan Adamus, Radosław Michalski, Adam Sikora

**Affiliations:** Institute of Applied Radiation Chemistry, Lodz University of Technology, Lodz, Poland

**Keywords:** Azanone, Nitroxyl, HNO, Thiols, Boronate probes

## Abstract

**HNO** (nitroxyl, IUPAC name azanone) is an electrophilic reactive nitrogen species of growing pharmacological and biological significance. Here, we present data on the pH-dependent kinetics of azanone reactions with the low molecular thiols glutathione and *N*-acetylcysteine, as well as with important serum proteins: bovine serum albumin and human serum albumin. The competition kinetics method used is based on two parallel **HNO** reactions: with **RSH**/**RS**^**−**^ or with **O**_**2**_. The results provide evidence that the reaction of azanone with the anionic form of thiols (**RS**^**−**^) is favored over reactions with the protonated form (**RSH**). The data are supported with quantum mechanical calculations. A comprehensive discussion of the **HNO** reaction with thiolates is provided.

## Introduction

**HNO** (nitroxyl, **IUPAC** name azanone) is the protonated product of the one-electron reduction of nitric oxide (^•^**NO**). In contrast to ^**•**^**NO**, azanone is a strong electrophile that is highly reactive toward various nucleophiles. It can also be oxidized to ^**•**^**NO** [***E*]*°***(^**•**^**NO**/**HNO**) = − 0.14 V [1], recently revised as ***E°***(^**•**^**NO**/**HNO**) = 0.27 V [2]. **HNO** reacts with molecular oxygen, [3–9], nitric oxide [10], nitrite [11, 12], hydroxylamine [12, 13], sulfite [12], thiosulfate [12, 13], metalloproteins [14–18], metalloporphyrines [19–22], thiols [3, 13, 14, 23], C- and S-nitroso compounds [12, 24], nitroxides [13, 25–27] and phosphines [12, 13, 28–33]. Thiols constitute the main biological target of **HNO** [34]. The fast reaction between **HNO** and thiols (~10^6^ M^−1^s^−1^ [3, 13, 14]) results in the formation of *N*-hydroxysulfenamide (Reaction 1), which in the presence of excess thiol is converted into the corresponding disulfide and hydroxylamine (Reaction 2). *N*-hydroxysulfenamide can also undergo spontaneous isomerization to sulfinamide (Reaction 3). It has been postulated that sulfinamides are unique products of the **HNO** reaction with thiols and might serve as in vivo biomarkers of azanone formation [34].

1 $${\mathrm{HNO}} + {\mathrm{RS}}^- \to {\mathrm{RSNHO}}^-\mathop{\longrightarrow}\limits^{{{\mathrm{H}}^ + }}{\mathrm{RSNHOH}}$$

2 $${\mathrm{RSNHOH}} + {\mathrm{RSH}} \to {\mathrm{RSSR}} + {\mathrm{NH}}_2{\mathrm{OH}}$$

3 $${\mathrm{RSNHOH}} \to {\mathrm{RS}}({\mathrm{O}}){\mathrm{NH}}_2$$

The high reactivity of **HNO** toward thiols and their abundance in biological systems are major factors determining the short lifetime of azanone in vivo [34]. On the other hand, it has been proposed that azanone can be generated in several thiol-related pathways [35, 36]. The first is the reaction of thiols with S-nitrosothiols (Reaction 4) [37, 38]. Similar routes of **HNO** generation include **RSNO** reactions with **H**_**2**_**S** (Reaction 5 and/or Reactions 6–7) [39] or ascorbate anion (**Asc**^**−**^) (Reactions 8–9) [40].

4 $${\mathrm{RSNO}} + {\mathrm{R}}^\prime {\mathrm{SH}} \to {\mathrm{RSSR}}^\prime + {\mathrm{HNO}}$$

5 $${\mathrm{RSNO}} + {\mathrm{HS}}^ - \to {\mathrm{RSS}}^ - + {\mathrm{HNO}}$$

6 $${\mathrm{RSNO}} + {\mathrm{HS}}^ - \to {\mathrm{RSH}} + {\mathrm{SNO}}^ -$$

7 $${\mathrm{SNO}}^ - + {\mathrm{HS}}^ - + {\mathrm{H}}^ + \to {\mathrm{HSS}}^ - + {\mathrm{HNO}}$$

8 $${\mathrm{RSNO}} + {\mathrm{Asc}}^ - \to {\mathrm{RSH}} + {\mathrm{AscNO}}^ -$$

9 $${\mathrm{AscNO}}^ - \to {\mathrm{DHA}} + {\mathrm{HNO}}$$

Doctorovich et al. demonstrated that azanone can also be formed during the reactions of ^**•**^**NO** with ascorbate or phenols (e.g., tyrosine, hydroquinone, salicylic acid, α-tocopherol or acetaminophen), according to Reaction 10 [41, 42].

10 $$^{\cdot }{\mathrm{NO}} + {\mathrm{ROH}} \to {\mathrm{RO}}^ \cdot + {\mathrm{HNO}}$$

Recently, it has been proposed that **HNO** is also produced in the reaction of thiols with nitric oxide [36]. ^**•**^**NO** is known to react with thiols, with the formation of **N**_**2**_**O** and corresponding disulfides [43] and/or sulfenic acids [44]. However, both the mechanism [36, 43, 44] and the kinetics of these processes are elusive [36, 45–47]. The formation of **HNO** has also been linked to the mechanism describing the formation of dinitrosyl-iron complexes (**DNIC**) from ^**•**^**NO**, **RS**^−^ and **Fe**^**2+**^ [48–50]. **DNIC** are biologically relevant bioinorganic complexes of ^**•**^**NO**, and perhaps the most abundant nitric oxide-derived adducts present in cells producing ^**•**^**NO** [51, 52]. It has been suggested that they can act as **RSNO** precursors [49, 50, 53] and **HNO**/**NO**^−^ donors [54, 55]. Due to the rapid scavenging of **HNO** by thiols, the generation of azanone is not expected to affect the **DNIC**-dependent **RSNO** formation. The number of these processes makes it challenging to formulate a proper description of the thiols/^**•**^**NO**/**HNO** interactome.

In the absence of scavengers, **HNO** is known to spontaneously dimerize with a second-order rate constant of ~8 × 10^6^ M^−1^s^−1^ [10]. The intermediate product of this reaction, hyponitrous acid, dehydrates to final decomposition products, nitrous oxide and water (Reaction 11).

11 $$2{\mathrm{HNO}} \to \left[ {{\mathrm{HONNOH}}} \right] \to {\mathrm{N}}_2{\mathrm{O}} + {\mathrm{H}}_2{\mathrm{O}}$$

The propensity of **HNO** to undergo the above reaction requires the use of donor molecules, the decomposition product of which is the **HNO** molecule. The most often studied and commonly used **HNO** donor is Angeli’s salt, which decomposes at 25 ˚C with a rate constant of 6.8 × 10^−4^ s^−1^ (t_1/2_ ~ 17 min) in a pH range from 4 to 8.6 [56, 57]. The fact that its decomposition rate constant is independent of pH is a unique feature of Angeli’s salt compared to other **HNO** donors. Other frequently used **HNO** donors are Piloty’s acid and its derivatives [57–61]. Unsubstituted Piloty’s acid (*N*-hydroxybenzenesulfonamide) releases azanone favorably under alkaline conditions only, whereas Piloty’s acid derivatives, substituted at different positions of the aromatic ring, release azanone across a wide range of pH values [57, 60, 61]. The rate constant of **HNO** release at a given pH depends on the ring substituents in Piloty’s acid derivatives [60, 61].

Similarly to ^•^**NO**, **HNO** exhibits unique pharmacological effects that have potential benefits for the treatment of a variety of diseases. Chronologically, the first described biological action of **HNO** was the inhibition of alcohol dehydrogenase by cyanamide (a pharmacological alcohol deterrent agent), *via* its catalase-dependent bioactivation into an **HNO** donor [62–64]. More recently, **HNO** donors have been proposed as agents for the treatment of heart failure [65–68]. Azanone donors have been shown to induce apoptosis, suppress tumor angiogenesis, and help to achieve analgesia [69–73]. Some of these effects could be connected to **HNO** reactions, mainly with cysteine residues of key enzymes responsible for the observed pharmacological effects. For instance, **HNO** generated from cyanamide modifies the cysteine-302 residue in aldehyde dehydrogenase, leading to irreversible inhibition of the enzyme [63]. The mechanism by which azanone affects the heart is a matter of intense research. It has been proposed that **HNO** donors enhance cardiac contractility, by targeting the regulatory protein phospholamban [74, 75]. Keceli et al. found that **HNO** reacts with Cys-41 and Cys-46 via the formation of the intramolecular disulfide bond, which forces conformational changes in the protein and enhances cardiac function as a result [74].

In a previous study, we investigated the reactivity of **HNO** toward selected thiols: cysteine (*k*_*Cys*_ = (4.5 ± 0.9) × 10^6^ M^−1^s^−1^, p*K*_a_ = 8.3), glutathione (*k*_*GSH*_ = (3.1 ± 0.6) × 10^6^ M^−1^s^−1^, p*K*_a_ = 8.8), *N*-acetylcysteine (*k*_*NAC*_ = (1.4 ± 0.3) × 10^6^ M^−1^s^−1^, p*K*_a_ = 9.5) and captopril (*k*_*Cap*_ = (6 ± 1) × 10^5^ M^−1^s^−1^, p*K*_a_ = 9.8). We found that at pH 7.4 the rate constant of the **HNO** reaction with thiol depends on its **–SH** group p*K*_a_. [3] In the present study, we explored the dependence of the rate constants of the reactions of **HNO** with selected biologically important thiols on pH. The data show the effect of pH on **HNO** reactivity toward the low molecular thiols *N*-acetylcysteine and glutathione and the thiol proteins bovine and human serum albumins.

## Materials and Methods

### Materials

Angeli’s salt (**AS**, Sodium Trioxodinitrate, **Na**_**2**_**N**_**2**_**O**_**3**_) was synthesized according to a published procedure [57]. Stable solutions of Angeli’s salt prepared in 1 mM NaOH (pH ∼11) were stored on ice during the experiments and each day a fresh **AS** stock solution was prepared [56, 57]. A boronate probe, coumarin boronic acid (**CBA**), which enables the detection of peroxynitrite (**ONOO**^**–**^) formed in the reaction of **HNO** with molecular oxygen, was synthesized according to a published procedure [76]. All thiols (glutathione (**GSH**), *N-*acetylcysteine (**NAC**), bovine serum albumin (**BSA**) and human serum albumin (**HSA**)), as well as all other chemicals (of the highest purity available) were purchased from Sigma-Aldrich Corp. By varying the amounts of salts (monobasic dihydrogen phosphate and dibasic monohydrogen phosphate) a range of buffers between pH 6.4 and 8.3 were prepared. All solutions were prepared using deionized water (Millipore Milli-Q system).

### Competition Kinetic Method

**Scheme 1:**
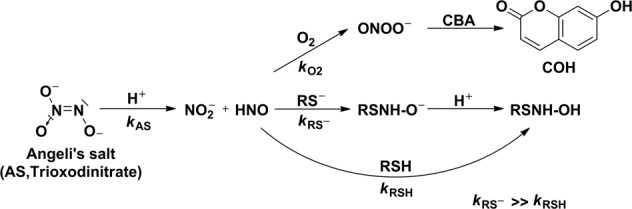
The reaction model used to determine the rate constants of the reactions of **HNO** with **RSH**/**RS**^**−**^

**Fig. 1:**
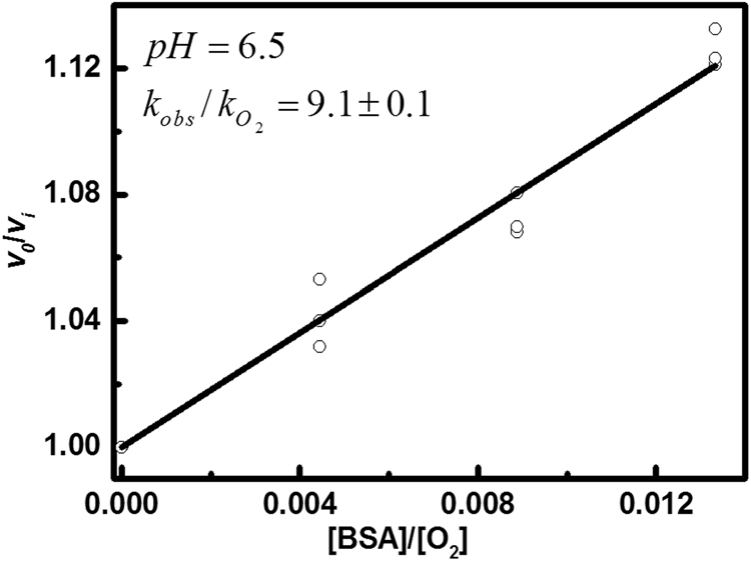
The *υ*_0_/*υ*_i_ ratio as a function of **[BSA]**/**[O**_**2**_**]** at pH = 6.5

The competition kinetic method used in this study followed the procedure described previously [3, 12]. Angeli’s salt, the most common **HNO** donor, was used. Azanone released from Angeli’s salt reacts either with a corresponding thiol (**RSH** and **RS**^**−**^) or with molecular oxygen (Scheme 1). The latter, relatively fast reaction (*k* = (1.8 ± 0.3) × 10^4^ M^−1^s^−1^) [3] results in the formation of peroxynitrite, which can be easily detected fluorometrically with the use of the fluorogenic probe, coumarin boronic acid (**CBA**). Across the whole studied pH range, **CBA** reacts rapidly and directly with **ONOO**^**–**^ (*k* = 7.3 × 10^5^ M^−1^s^−1^, pH 6.6; *k* = 1 × 10^6^ M^−1^s^−1^, pH 7.4; *k* = 4.5 × 10^5^ M^−1^s^−1^, pH 8.2), with the formation of blue fluorescent 7-hydroxycoumarin (**COH**) as the main product [3, 76]. The ratio of initial rates of **COH** formation in the absence and presence of scavenger S can be expressed by the equation

12 $$\frac{{v_0}}{{v_{\mathrm{i}}}} = 1 + \frac{{k_{{\mathrm{obs}}}\left[ {\mathrm{S}} \right]_{\mathrm{i}}}}{{k_{{\mathrm{O}}2}\left[ {{\mathrm{O}}_2} \right]}}$$

where ***k***_**obs**_ and ${\boldsymbol{k}}_{{\mathbf{O}}_{\mathbf{2}}}$ are the second order rate constants of **HNO** reactions with the scavenger (thiol/thiolate) and molecular oxygen, respectively, and [S] and [O_2_] denote the total concentrations of thiol ([**S**] = [**RSH**] + [**RS**^**−**^]) and molecular oxygen. In solutions remaining in equilibrium with air the concentration of molecular oxygen is equal to 225 μM [77]. Based on Eq. (1), the ***k***_**obs**_/${\boldsymbol{k}}_{{\mathbf{O}}_{\mathbf{2}}}$ ratio was determined for each pH value. Figure 1 illustrates the used method to determine the ***k***_**obs**_/${\boldsymbol{k}}_{{\mathbf{O}}_{\mathbf{2}}}$ ratio at pH 6.5.

### Stopped-flow Measurements

Angeli’s salt (6 μM in 1 mM NaOH) was mixed rapidly with a solution containing the coumarin based monoborate probe **CBA** (50 μM), phosphate buffer (50 mM, pH range 6.4–8.3), metal chelator **dtpa** (100 μM), 10% **CH**_**3**_**CN** and the corresponding thiol compound at the appropriate concentration. Glutathione and *N*-acetylcysteine were used in a concentration range from 1 to 3 μM. Human or bovine serum albumin were used in the concentration range from 2 to 6 μM. Both reaction mixtures - the alkaline solution of the **HNO** donor and the solution of the corresponding thiol in the appropriate phosphate buffer - remained in equilibrium with air. The formation of fluorescent **COH** was monitored using an Applied Photophysics SX20 stopped-flow spectrophotometer equipped with a fluorescence detector and a thermostatically controlled cell (25 °C) with a 10-mm optical pathway. The reaction mixtures were excited at 332 nm and the emitted light intensity was measured at 470 nm (PMT voltage = 850 V, emission/excitation slit = 2.5 nm). The initial rates of the increase in the fluorescence intensity were fitted with a linear function. The data were analyzed using the Origin Pro 2015 program (OriginLab Corporation, Northampton, MA, USA).

### pH Determination

The pH of the phosphate buffers and the exact pH of the solutions after mixing were measured using a SevenMulti^TM^ pH meter (Mettler Toledo GmbH, Schwerzenbach, Switzerland).

### Computational Details

Quantum mechanical calculations were performed in the Gaussian G09 suite of programs, Revision E01 [78]. The geometries of the stationary points were fully optimized using the Hartree-Fock (HF) method as well as Density Functional Theory (DFT). The functionals M06-2X [79], B2PLYP and B3LYP were used with Grimme’s D3 dispersion correction, B2PLYP-D3 [80–82] and B3LYP-D3 [83, 84], respectively. A 6–311++G(2df,2p) basis set [85] was used, with the inclusion of a water solvent. Water was represented according to the IEFPCM [86, 87] method by a default polarizable continuum solvent model in Gaussian. Structure optimizations were followed by frequency calculations, in order to verify the nature of the stationary points and to obtain corresponding free energy values. We were unable to locate any transition states for the reaction between **CH**_**3**_**SH** and **CH**_**2**_**O**.

## Results

To examine the effect of pH on the reactivity of azanone toward thiols, we used the competition kinetics method previously described in the literature [3, 12]. Due to the spontaneous dimerization of azanone, donor compounds are required that decompose with the release of the **HNO** molecule [10]. In our study, Angeli’s salt was used as the **HNO** donor because it decomposes with a constant rate in the pH range from 4 to 8.6 [56, 57]. Above pH 8, the rate of decomposition decreases [56, 57]. The low concentration of the donor compound (3 μM) in the system resulted in an initial flux of **HNO** below 0.15 μM/min. Therefore, the steady-state concentration of **HNO** was very low. In the entire considered pH range, the system remained in equilibrium with air. Hence, the concentration of molecular oxygen was equal to 225 μM [77]. Given all the above-mentioned factors, the **HNO** dimerization process was negligible and was not taken into account.

Azanone is a weak acid, with a p*K*_*a*_ value of 11.4 [1]. Therefore, released azanone exists in its protonated form in the studied pH range. The rate constant of the **HNO** reaction with molecular oxygen had been determined previously as equal to (1.8 ± 0.3) × 10^4^ M^−1^ s^−1^. We assume that this rate constant does not depend on the pH [3]. The reaction between **HNO** and **O**_**2**_ results in the formation of peroxynitrite (ONOO^−^) [3], which in aqueous solutions exists in an acid-base equilibrium with its protonated form peroxynitrous acid (**ONOOH**, p*K*_*a*_ = 6.8) [88]. In the absence of scavengers, peroxynitrite undergoes isomerization to HNO_3_ (~70%) (Reaction 12) and homolysis to ^•^OH and ^•^NO_2_ radicals (~30%) (Reaction 13). All these radical species are highly oxidizing and nitrating agents. Its formation in the presence of the **HNO** donor could lead to one-electron oxidation of the donor compound, affecting the kinetics and mechanism of its decay [61]. The use of boronate probes in the system helps effectively scavenge peroxynitrite and prevent oxidation of Angeli’s salt [61].

13 $${\mathrm{ONOOH}} \to {\mathrm{NO}}_3^ - + {\mathrm{H}}^ +$$

14 $${\mathrm{ONOOH}} \to ^{\, {\mathbf{\cdot}} }{\mathrm{NO}}_2 + ^{\, \cdot }{\mathrm{OH}}$$

The oxidation of boronate compounds by **ONOO**^**−**^ is a direct, stoichiometric and rapid reaction (*k* ~ 10^5^–10^6^ M^−1^s^−1^), leading to the formation of the corresponding phenols as major products [76, 89–93]. However, at pH higher than 9 boronates undergo an addition reaction with hydroxyl ions (**HO**^**–**^), yielding a product unreactive toward **ONOO**^**–**^ [91]. Given the pH-dependence of boronates reactivity toward **ONOO**^**−**^ and the lower release of **HNO** from Angeli’s salt in alkaline solutions (pH > 8.6), our studies were performed in a limited pH range (6.4–8.3) [56, 57]. The probe used in our study, coumarin boronic acid (**CBA**), is converted by peroxynitrite to blue fluorescent 7-hydroxycoumarin (**COH**) as a major product [76]. The high reactivity of **CBA** toward peroxynitrite within the studied pH range ensures quantitative peroxynitrite scavenging in the presence of low micromolar concentrations of the studied thiols. The formation of **COH** formation in the presence of thiols was slower than in their absence. Based on Eq. (1), we determined the ratios of the second-order rate constants of the **HNO** reactions with thiol and molecular oxygen for each pH in the range from 6.4 to 8.3.

Assuming that **HNO** can react with the thiolate anion **RS**^**–**^ (***k***_***RS***_***-***) as well as with its protonated form **RSH** (***k***_***RSH***_), the observed rate constant (***k***_***obs***_) can be expressed as a function of pH, which depends on thiol p*K*_*a*_, and the rate constants ***k***_***RS***_***-*** and ***k***_***RSH***_:

15 $$k_{obs} = \frac{{k_{RS^ - } \cdot 10^{ - pk_a} + k_{RSH} \cdot 10^{ - pH}}}{{10^{ - pk_a} + 10^{ - pH}}}$$

The ratio ***k***_***obs***_ /${\boldsymbol{k}}_{{\mathbf{O}}_{\mathbf{2}}}$ can be expressed in a similar way:

16 $$k_{obs}/k_{O_2} = \frac{{k_{RS^ - } \cdot 10^{ - pk_a} + k_{RSH} \cdot 10^{ - pH}}}{{\left( {10^{ - pk_a} + 10^{ - pH}} \right) \cdot k_{O_2}}}$$

**Fig. 2:**
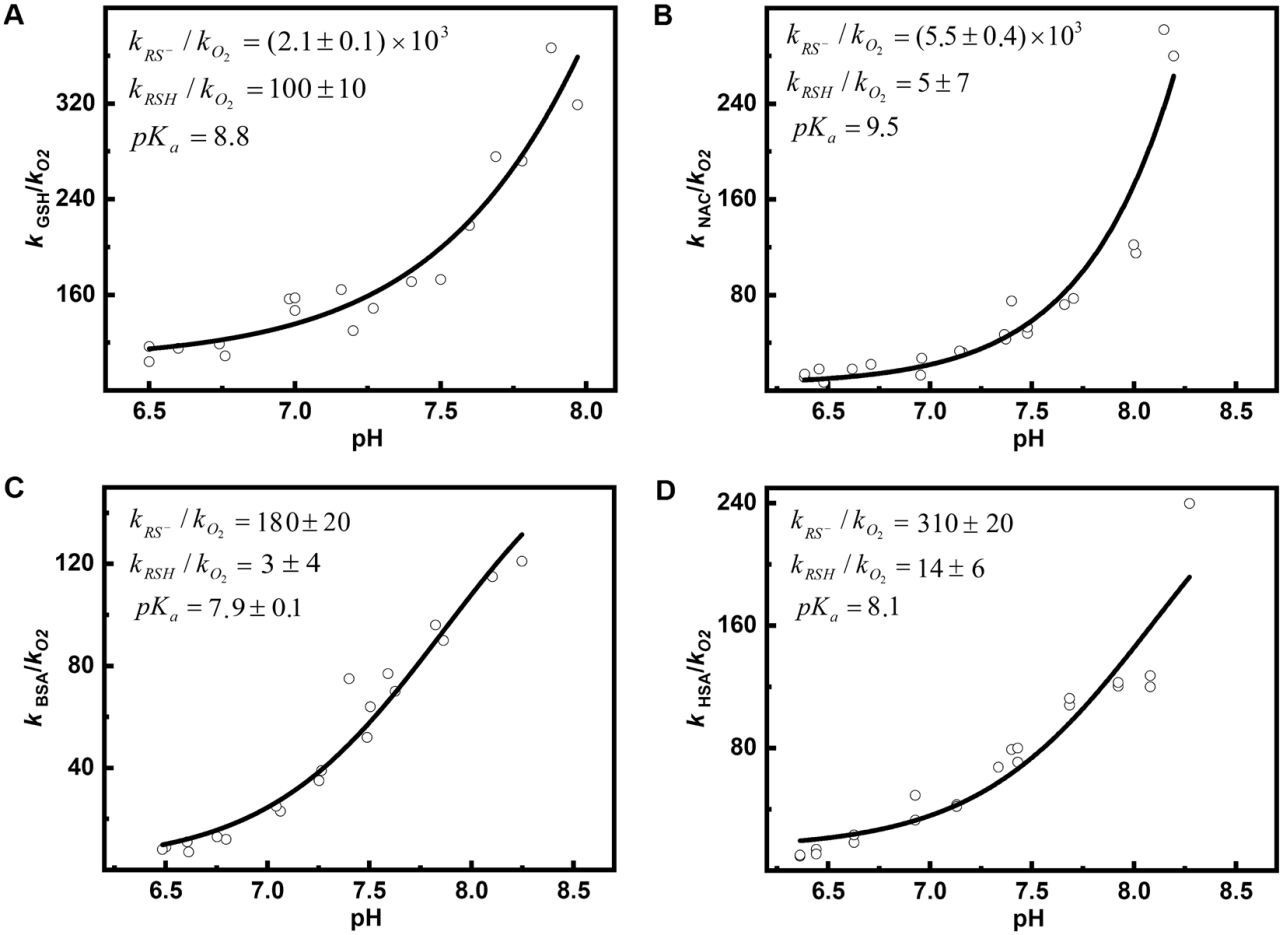
The ***k***_***obs***_/${\boldsymbol{k}}_{{\mathbf{O}}_{\mathbf{2}}}$ ratio as a function of pH. **A** Glutathione (**GSH**), **B** *N*-acetylcysteine (**NAC**), **C** Bovine serum albumin (**BSA**), **D** Human serum albumin (**HSA**). Reaction mixtures consisted of 0; 0,5; 1; 1,5 μM **GSH** or **NAC** or 0; 1; 2; 3 μM **BSA** or **HSA**, 25 μM coumarin boronic acid (**CBA**), 3 μM Angeli’s salt (**AS**) in phosphate buffers (25 mM) at appropriate pH with addition of **dtpa** (50 μM) and 5% **CH**_**3**_**CN**. The solutions were excited at 332 nm, the emitted light intensity was measured at 470 nm (photomultiplier voltage: 850 V, emission/excitation slit: 2.5 nm)

The ***k***_***obs***_ /${\boldsymbol{k}}_{{\mathbf{O}}_{\mathbf{2}}}$ ratios obtained for different pH were fitted to Eq. (3), which allowed us to estimate the rate constants of the **HNO** reaction with the corresponding thiol and thiolate, separately. Figure 2A shows the dependence of the ***k***_**obs**_/${\boldsymbol{k}}_{{\mathbf{O}}_{\mathbf{2}}}$ ratio on pH for the reaction between **HNO** and glutathione. It is noticeable that the reactivity of **HNO** toward thiols is pH-dependent. The p*K*_*a*_ value for the dissociation of the **-SH** group in glutathione was taken from the literature as being equal to 8.8 [94]. The best fitting was obtained assuming ***k***_***RS***_***-*** /${\boldsymbol{k}}_{{\mathbf{O}}_{\mathbf{2}}}$ equal to (2.1 ± 0.1) × 10^3^ and ***k***_**RSH**_/${\boldsymbol{k}}_{{\mathbf{O}}_{\mathbf{2}}}$ = 100 ± 10. These results indicate that thiolate anions are much more reactive toward **HNO** than their protonated forms (***k***_***RS***_***-*** » ***k***_**RSH**_). A similar observation was made for *N-*acetylcysteine (Fig. 2B). The p*K*_*a*_ value of the **-SH** group in *N-*acetylcysteine is equal to 9.5 [94] and the corresponding ratios ***k***_***RS***_***-****/*${\boldsymbol{k}}_{{\mathbf{O}}_{\mathbf{2}}}$ and ***k***_**RSH**_/${\boldsymbol{k}}_{{\mathbf{O}}_{\mathbf{2}}}$ are equal to (5.5 ± 0.4) × 10^3^ and 5 ± 7, respectively. Again, the reaction of **HNO** with thiolate is faster, hence favored.

We also performed analogical experiments for the two most abundant thiol proteins, bovine (**BSA**) and human (**HSA**) serum albumins. The p*K*_*a*_ values of these proteins are debatable. In the literature, the p*K*_*a*_ value of **BSA** cysteine **-SH** group is estimated to be in the range from 7.86 to 8.00 [95], whereas the spectrum of p*K*_*a*_ values for **HSA** is even broader (5.0–8.8) [96–101]. Figure 2C shows the dependence of the ***k***_**obs**_/${\boldsymbol{k}}_{{\mathbf{O}}_{\mathbf{2}}}$ ratio on pH for the reaction between **HNO** and **BSA**. The experimental data are best fitted with Eq. (3) and give a p*K*_*a*_ value for the dissociation of the **-SH** group in **BSA** equal to 7.9 ± 0.1. The obtained p*K*_*a*_ value fits well into the range of values described in the literature. The ratios ***k***_***RS***_***-***/${\boldsymbol{k}}_{{\mathbf{O}}_{\mathbf{2}}}$ and ***k***_**RSH**_/${\boldsymbol{k}}_{{\mathbf{O}}_{\mathbf{2}}}$ are equal to 180 ± 20 and 3 ± 4, respectively. Figure 2D shows the dependence of the ***k***_**obs**_/${\boldsymbol{k}}_{{\mathbf{O}}_{\mathbf{2}}}$ ratio on pH for the reaction between **HNO** and **HSA**. In calculations performed for **HSA**, we used a value for p*K*_*a*_ of 8.1 [101], as has been recently established by three independent approaches. The experimental data, best fitted with Eq. (3) assuming the above-mentioned p*K*_*a*_ value for the dissociation of the **-SH** group in **HSA**, give the ratios ***k***_***RS***_***-***/${\boldsymbol{k}}_{{\mathbf{O}}_{\mathbf{2}}}$ = 310 ± 20 and ***k***_**RSH**_/${\boldsymbol{k}}_{{\mathbf{O}}_{\mathbf{2}}}$ = 14 ± 6. The ratio values obtained for the thiol proteins confirm the observed relationship between **HNO** reactivity and protonation of the sulfhydryl group in the studied compounds.

**Fig. 3:**
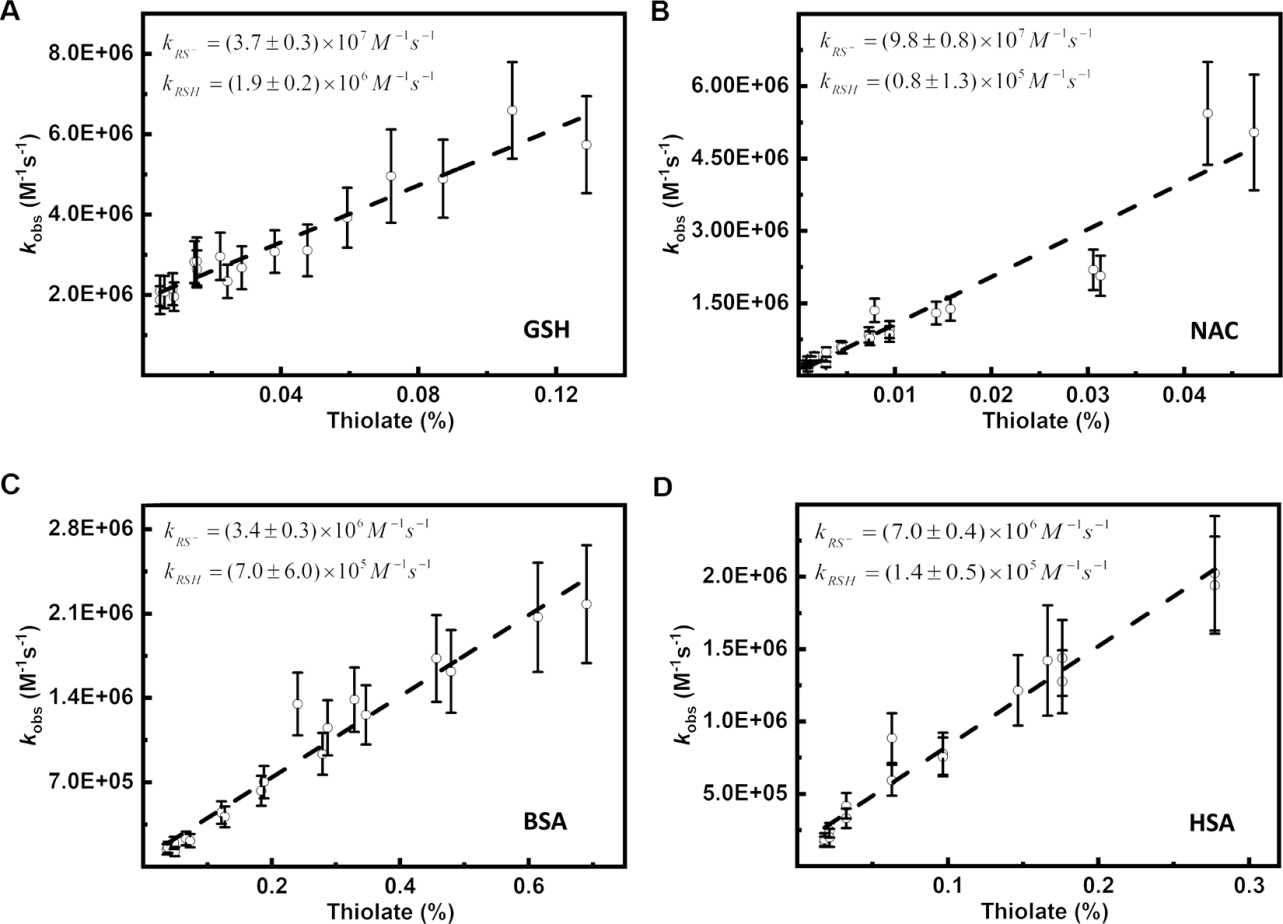
Correlation between the observed rate constant (***k***_***obs***_) and the deprotonated form of (**A**) Glutathione (**GSH**), **B** *N*-acetylcysteine (**NAC**), **C** Bovine serum albumin (**BSA**), **D** Human serum albumin (**HSA**). The concentration of the deprotonated form of each thiol was assessed using the appropriate p*K*_a_ value: **A** p*K*_a_ = 8.8 [94], **B** p*K*_a_ = 9.5 [94], **C** p*K*_a_ = 7.9 (this work), **D** p*K*_a_ = 8.1 [101]. The appropriate ***k***_***obs***_ was calculated from the experimentally obtained ratio ***k***_***obs***_/${\boldsymbol{k}}_{{\mathbf{O}}_{\mathbf{2}}}$ using ${\boldsymbol{k}}_{{\mathbf{O}}_{\mathbf{2}}}$ = (1.8 ± 0.3) × 10^4^ M^−1^ s^−1^ [3]. Reaction mixtures consisted of 0; 0,5; 1; 1,5 μM **GSH** or **NAC** or 0; 1; 2; 3 μM **BSA** or **HSA**, 25 μM coumarin boronic acid (**CBA**), 3 μM Angeli’s salt (**AS**) in phosphate buffers (25 mM) at appropriate pH with addition of **dtpa** (50 μM) and 5% **CH**_**3**_**CN**. The solutions were excited at 332 nm, the emitted light intensity was measured at 470 nm (photomultiplier voltage 850 V, emission/excitation slit 2.5 nm)

To further examine the reaction of **HNO** with thiolates, we analyzed the correlation between ***k***_**obs**_ and the thiolate concentration. The values for ***k***_***obs***_ were determined based on the rate constant of the **HNO** reaction with molecular oxygen ${\boldsymbol{k}}_{{\mathbf{O}}_{\mathbf{2}}}$ = (1.8 ± 0.3) × 10^4^ M^−1^s^−1^ [3], whereas the concentration of thiolate was calculated based on the corresponding p*K*_*a*_ value of the thiol. The linear relationship between ***k***_**obs**_ and the thiolate concentration can be expressed by Eq. 4. The correlation is illustrated in Fig. 3.

17 $$k_{obs} = \frac{{\left[ {RS^ - } \right]}}{{[S]}} \cdot \left( {k_{RS^ - } - k_{RSH}} \right) + k_{RSH}$$

The variables [**RS**^–^] and [**S**] denote the concentration of thiolate and the total concentrations of the thiol ([**S**] = [**RSH**] + [**RS**^**−**^]), respectively, while **k**_**RS**−_ or **k**_**RSH**_ are the rate constants of the **HNO** reaction with the thiolate (**RS**^−^) and thiol (**RSH**), respectively. Therefore, our approach also allows us to estimate the rate constants of the **HNO** reaction with the corresponding thiol and thiolate, separately. The rate constants of the **HNO** reaction with the appropriate thiolates is high and varies in the range *k* ~10^6^ – 10^7^ M^−1^s^−1^, whereas the rate constants of the **HNO** reaction with the respective thiols is an order of magnitude lower at *k* ~10^5^ – 10^6^ M^−1^s^−1^ (Table 1). Therefore, the same tendency can be observed: the reaction between azanone and thiolate is favored. There is a slight discrepancy between the ***k***_**RSH**_ values for thiol proteins computed with the aid of each approach. As can be seen in Fig. 3, the thiolate percentage is strongly dependent on the p*K*_*a*_ value, which according to the literature varies in the case of **BSA** and **HSA** [95–101].

**Table 1:** Comparison of the rate constants of the HNO reaction with protonated and deprotonated forms of the studied thiols based on two different approaches: (a) based on pH dependence of the *k*_*obs*_/${\boldsymbol{k}}_{{\mathbf{O}}_{\mathbf{2}}}$ ratio presented in Fig. 2; (b) based on the correlation between *k*_*obs*_ and the thiolate concentration of the appropriate thiol presented in Fig. 3

Thiol	^a^*k*_*RS*_- (M^−1^s^−1^)	^a^*k*_*RSH*_ (M^−1^s^−1^)	^b^*k*_*RS*_- (M^−1^s^−1^)	^b^*k*_*RSH*_ (M^−1^s^−1^)
**GSH**	(3.8 ± 0.8) × 10^7^	(1.8 ± 0.5) × 10^6^	(3.8 ± 0.3) × 10^7^	(1.9 ± 0.2) × 10^6^
**NAC**	(9.9 ± 2.4) × 10^7^	(0.9 ± 1.4) × 10^5^	(9.8 ± 0.8) × 10^7^	(0.8 ± 1.3) × 10^5^
**BSA**	(3.2 ± 0.9) × 10^6^	(5.4 ± 8.1) × 10^4^	(3.4 ± 0.3) × 10^6^	(7.0 ± 6.0) × 10^5^
**HSA**	(5.6 ± 1.3) × 10^6^	(2.5 ± 1.5) × 10^5^	(7.0 ± 0.4) × 10^6^	(1.4 ± 0.5) × 10^5^

To confirm our findings, quantum mechanical calculations were performed using different computational methods (Tables 2, 3). Different calculation methods for estimation of the energy barrier in the reaction of methyl thiolate (**MeS**^−^) with **HNO** give discrepant values. The relatively high energy barrier (125 kJ/mol) was computed with ab initio HF theory, whereas DFT calculations predicted the barrier to be about 27 kJ/mol for the M06-2X DFT functional and 13, 12 kJ/mol for the B2PLYP-D3 and B3PLYP-D3 functionals, respectively. It is worth underlining that the energy barrier calculated for the reaction of **HNO** with **MeS**^**−**^ is about 130–150 kJ/mol lower than the corresponding energy barrier estimated for the reaction of neutral reactants, i.e., **HNO** and **MeSH**. The 130 kJ/mol difference was computed based on HF theory, while DFT calculations estimated the difference as about 136 kJ/mol for the M06-2X DFT functional and 145, 148 kJ/mol for the B2PLYP-D3 and B3PLYP-D3 DFT functionals, respectively. It is interesting that even though the absolute energies computed for stationary points using DFT methods were significantly lower than the values obtained with HF, they resulted in slightly higher energy differences. Nevertheless, the qualitative outcomes of the different theoretical calculations were consistent, showing that azanone reacts faster with the thiolate. In the reverse reaction, ~185–200 kJ/mol more energy is required for **MeSNHOH** decomposition than for decomposition of its deprotonated form **MeSNHO**^**–**^ into substrates, i.e., **HNO** and the thiolate. The calculated energy barriers for the reaction of methyl thiolate (**MeS**^**−**^) with electrophilic **CH**_**2**_**O** (isoelectric with **HNO**) are similar in value to those calculated for the **MeS**^**−**^ reaction with **HNO** (Table 2).

**Table 2:** Comparison of computationally evaluated Gibbs free energies (ΔG^‡^) of the studied reactions obtained using various theoretical methods

Reaction	ΔG^‡^(kJ/mol)			
	HF	M06-2X	B2PLYP-D3	B3LYP-D3
**HNO+MES**^−^ → **MeSNHO**^−^	125.0	26.7	12.7	11.7
**HNO+MESH** → **MeSNHOH**	257.1	163.3	158.2	159.8
**CH**_**2**_**O+MeS**^**−**^ → **MeSCH**_2_**O**^−^	101.4	29.8	28.0	–^a^
**MeSNHO**^−^ → **HNO+MeS**^−^	45.4	16.6	3.3	2.1
**MeSNHOH** → **HNO+MeSH**	245.5	204.9	187.4	187.6
**MeSCH**_**2**_**O**^**−**^ → **CH**_**2**_**O**+**MeS**^−^	0.2	1.6	0.8	–

^a^Lack of data due to no transition state found

## Discussion

The mechanism of the **HNO** reaction with thiols is currently understood to involve an initial nucleophilic attack of the thiol on the electrophilic nitrogen of azanone, forming *N*-hydroxysulfenamide (Reaction 1) [34, 35]. Therefore, our finding that the rate constant of the reaction of **HNO** with thiols depends on its **p*K***_**a**_ value is not surprising. Using the presented approaches, we were able to estimate the rate constants of the **HNO** reaction with the corresponding thiol and thiolate separately. Supported by quantum mechanical calculations, the results indicate that azanone is much more reactive toward thiolates (**RS**^**−**^) than toward protonated forms of thiols (**RSH**). This leads to the conclusion that it is the thiolate that nucleophilically attacks the **HNO** double bond. This mechanism is similar to the well-established reaction mechanism of thiols with carbonyl compounds, including **CH**_**2**_**O**, leading to the formation of hemithioacetals. It is commonly accepted that in these reactions the addition of thiols proceeds by the reaction of thiolate anion **RS**^**—**^ [102, 103].

The mechanism of the reaction between azanone and thiols may be comparable to the formaldehyde (**CH**_**2**_**O**) reaction with thiols, in which formaldehyde acts as an electrophile that reacts with biological nucleophiles, including thiols [104]. By analogy, the first step of the reaction may be a nucleophilic attack by the thiolate on the **CH**_**2**_**O** double bond, leading to the formation of the *S*-hydroxymethyl adduct, an analog of *N*-hydroxysulfenamide [104].

Quantum mechanical results obtained for the detachment of **HNO** from **MeSNHO**^**–**^**/ MeSNHOH** can be compared with our recently published data on the decomposition of Piloty’s acid (*N*-hydroxybenzenesulfonamide, C_6_H_5_SO_2_NHOH) and its derivatives [61]. The mechanisms in the processes are quite similar. The decomposition mechanism of Piloty’s acid and its derivatives include initial deprotonation of oxygen (C_6_H_5_SO_2_NHO^**−**^) and subsequent S–N bond heterolysis, leading to slow release of the products—benzenesulfinate and **HNO** [57, 61]. We hypothesize that the p*K*_a_ value of **RSNHOH** at physiological pH may be high, so the protonation reaction of **RSNHO**^**–**^ occurs spontaneously. As a consequence, the stable **RSNHOH** is formed.

**Table 3:** Comparison of calculated bond lengths and selected angles for substrates, transition states and products of the studied azanone reactions with MeS^−^ or MeSH

	Bond length (Å)			Angle (°)		
	C-S	S-N	N-O	H-C-S	C-S-N	S-N-O
**MeS**^**—**^ **+ HNO** → **MeSNHO**^**—**^						
**HNO**						
HF	–	–	1.168			
M06-2X	–	–	1.192			
B2PLYP-D3	–	–	1.210			
B3LYP-D3	–	–	1.201			
**MeS**^**–**^						
HF	1.833	–	–			
M06-2X	1.836	–	–			
B2PLYP-D3	1.842	–	–			
B3LYP-D3	1.849	–	–			
**TS**						
HF	1.808	2.139	1.261	110.58	99.35	111.34
M06-2X	1.796	2.198	1.275	111.39	94.77	110.23
B2PLYP-D3	1.804	1.999	1.336	110.71	97.26	111.69
B3LYP-D3	1.812	2.017	1.329	110.56	98.46	111.99
**MeSHNO**^**–**^						
HF	1.807	1.691	1.383	106.79	106.16	115.25
M06-2X	1.812	1.726	1.391	107.77	104.01	114.19
B2PLYP-D3	1.820	1.749	1.404	107.62	104.86	114.54
B3LYP-D3	1.830	1.762	1.396	107.40	105.96	115.19
**MeSH + HNO** → **MeSNHOH**						
**HNO**						
HF	–	–	1.168			
M06-2X	–	–	1.192			
B2PLYP-D3	–	–	1.210			
B3LYP-D3	–	–	1.201			
**MeSH**						
HF	1.815	–	–			
M06-2X	1.816	–	–			
B2PLYP-D3	1.823	–	–			
B3LYP-D3	1.829	–	–			
**TS**						
HF	1.792	1.686	1.408	108.36	102.60	99.08
M06-2X	1.791	1.708	1.411	107.28	99.90	100.12
B2PLYP-D3	1.796	1.723	1.429	108.72	100.19	99.87
B3LYP-D3	1.805	1.728	1.427	108.16	100.67	99.95
**MeSNHOH**						
HF	1.805	1.681	1.385	105.75	105.37	115.94
M06-2X	1.809	1.693	1.412	105.89	104.24	114.99
B2PLYP-D3	1.816	1.699	1.435	105.64	105.26	115.21
B3LYP-D3	1.824	1.702	1.435	105.45	106.01	115.84

## Conclusion

The reaction of azanone with thiol proteins is one of the major factors responsible for its unique pharmacological effects. In the present study, we have demonstrated both that this reaction depends strongly on pH and that **HNO** is highly reactive toward thiolates (**RS**^−^). These results support the currently proposed reaction mechanism of **HNO** with thiols, involving an initial nucleophilic attack by the thiol on the electrophilic nitrogen of azanone.
